# Brooke–Spiegler Syndrome: Familial Cylindromatosis, a Rare Variant of a Rare Familial Syndrome

**DOI:** 10.1155/2021/7118260

**Published:** 2021-06-23

**Authors:** Harsh Patel, William Naber, Austin Cusick, Craig Oser

**Affiliations:** ^1^Ohio University, Heritage College of Osteopathic Medicine, 191 W Union St, Athens, OH 45701, USA; ^2^Riverside Methodist Hospital, 3535 Olentangy River Rd., Columbus, OH 43214, USA; ^3^Oser Plastic Surgery and Med Spa, 1 Robinson Plaza Suite 230, Pittsburgh, PA 15205, USA

## Abstract

Brooke–Spiegler Syndrome (BSS) is a rare autosomal dominant familial disorder resulting in dermatologic neoplasms of copious nodular appendages. Here, we report a case of Familial Cylindromatosis (FC), a subtype of BSS, in a patient with the largest cylindroma of 7.4 × 5.6 × 3.8 cm on the scalp. The patient had undiagnosed cylindromas growing for 36 years at presentation; however, he did not seek out healthcare evaluation. Excision and pathologic investigation of three large masses from different body sites determined a shared phenotype of cylindromas. Subsequent evaluation of the patient's son separately, after primary patient excision, confirmed cylindroma development as well. The pathologic evidence of cylindromas in the patient with a new history of family incidence confirmed the diagnosis of the FC variant of BSS.

## 1. Introduction

Brooke–Spiegler Syndrome (BSS), aptly named after its first descriptors in the late 1800s, is a rare autosomal dominant familial disorder with an unknown incidence and prevalence, resulting in dermatologic neoplasms of copious nodular appendages [1]. This condition first presents as 0.5 to 3.0 cm head nodules in adolescence with a 6–9.6 : 1 female to male ratio and has three common adnexal neoplasms: spiradenoma, cylindroma, and trichoepithelioma [1]. The Multiple Familial Trichoepitheliomas (MFT), formally Brooke–Foredyce Trichoepitheliomas, and Familial Cylindromatosis (FC), formally Ancell–Spiegler Cylindromas, are considered two distinct variants consisting only of their predominate histological subtype [2].

The FC and MFT subtypes are associated with CYLD gene mutations on chromosome 16q12-913 [2]. This tumor suppressor gene impedes the Tumor Necrosis Factor-alpha pathway by reducing the expression of nuclear factor kappa B (NF-kB), a transcription factor with a key role in the antiapoptotic process [2, 3]. BSS, FC, and MFT variants have simultaneously presented in patients, suggesting these seemingly distinct subtypes are actually the end result of phenotypic variability within the same disease.

Creating an effective treatment plan with careful follow-up is imperative as malignant transformation occurs in 5–10% of patients with BSS [1]. Considering the malignancy risk with inadequate treatment, we present a case of FC found in a father and son of a rural medically underserved community with poor medical accessibility. The patients cited their poor healthcare access delayed the evaluation of their conditions substantially. From this novel case, we demonstrate a successful treatment modality for substantial BSS masses and highlight the importance of proper medical care in underserved areas.

## 2. Case Presentation

**Figure 1:**
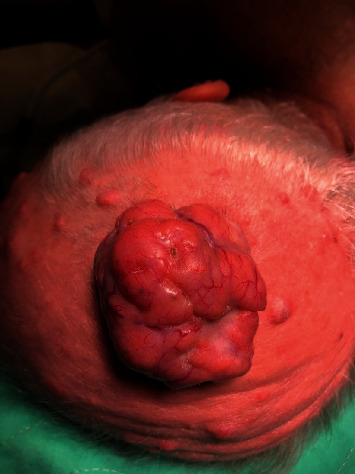
Cylindroma presentation on the patient's head, before surgical excision.

A 54-year-old male presented to the office with several subcutaneous nodular masses ranging in size on the scalp, groin, and back. The patient reports he had these nodules at age 18, but they have slowly grown over the years. The largest nodules were selected for surgical excision from each region. The nodules removed included a scalp lesion (7.4 × 5.6 × 3.8 cm) (Figure 1), right groin (4.7 × 2.7 × 1.9 cm), and upper back (4.4 × 3.5 × 3.2 cm). After excision, the three nodules had a similar gross appearance, gray-tan, firm, nodular mass. Scalp and back lesions were sent for frozen sectioning during the procedure but were inconclusive on the specific pathology. Frozen sectioning did raise concern for possible malignancy. Permanent sectioning revealed an irregular globular jigsaw of basaloid cell morphology of the dermis in all specimens. Hyaline droplets and focal ductal differentiation were also present in all specimens. No nuclear pleomorphism or mitotic elements were present. The tumor cells were positive for cytokeratin 7 (CK7), periodic acid-Schiff (PAS), and epithelial membrane antigen (EMA). These histologic findings were consistent with multiple cylindromas. The presence of multiple cylindromas raised concern for potential familial diseases such as Brooke–Spiegler syndrome. On follow-up, the patient discussed that his son had similar appearing nodules. On follow-up, the patient reported his son also had subsequent surgical resection. His lesions were determined to be cylindromas as well, solidifying a familial component.

## 3. Discussion

Brooke–Spiegler Syndrome is associated with a mutation of CYLD, a tumor suppressor gene. The gene codes for a deubiquitinating enzyme by removing the lysine 63-linked polyubiquitin chain from substrates. Subsequently, it impedes the NF-kB and c-Jun N-terminal kinase pathways [4]. The majority of the mutations are frameshift, nonsense, and missense, which lead to truncated proteins. Genetic evaluation of patients presenting with the classic BSS phenotype demonstrates germline CYLD mutation in 80–85% of cases, while the MFT phenotype demonstrates a germline mutation in 40–45% cases [4]. Interestingly, the clinical severity of the disease does not correlate with the genotype, and the phenotypic expression is variable between subsequent generations with the same germline mutations [4].

Cylindromas are usually benign slow-growing tumors presenting as red or pink dermal nodules with arborizing telangiectasias [5]. Histologically, cylindromas and spiradenomas are categorized as follicular tumors specifically derived from hair follicle bulge [6]. Cylindroma is comprised of tumor isles that are surrounded by a hyaline eosinophilic sheath. Within each island, there is an outer layer of poorly differentiated epithelial tumor cells with small dark nuclei along with well-differentiated inner cells with big pale nuclei [7]. Historically, both cylindromas and spiradenomas have been considered driven from an eccrine origin, but this has been recently questioned with new histochemistry techniques. Recent research shows that both tumors are found to be CD200 positive, an immunoprotective membranous molecule. CD200 is highly specific for the hair follicle bulges, and it further supports the common locations of these tumors on the scalp and face [6].

Evaluation and successful diagnosis is important as to rule out other syndromic pathologies and possible malignant potential. Syndromic considerations for a differential diagnosis included neurofibromatosis, basal cell nevus syndrome, toxic-exposure-related incidence, and tuberous sclerosis. The differential diagnosis of a particular lesion should include benign lesions such as those shared in the abovementioned syndromic disease presentation; however, due to size and uniqueness in the removed lesions, malignant cylindromas were to be considered.

Due to this condition's rarity, treatment modalities vary. Usually, surgical interventions, such as electro cryosurgery, laser surgery, and radiosurgery, have all been utilized [1]. Besides the surgical approach to treatment, medications including aspirin, adalimumab, topical imiquimod, and vismodegib have been trialed with varying success [1]. In 2016, Mulder et al. reported the use of intralesional triamcinolone acetonide injection as a treatment modality for cylindromas. They hypothesized that corticosteroids would lead to regression of the tumor since it suppresses the NF-kB pathway. Clinically, this treatment resulted in initial regression of the tumors, but the neoplasms began to regrow after a couple of months [8]. Further investigation of medical management could provide alternative treatments for patients with economic challenges.

We report this case to add to the incidence report of this rare variant of the rare syndrome. Moreover, we also want to highlight that our patient had cylindromas beyond his head and neck area including significantly large cylindromas in the upper back and groin. The largest cylindroma on the head measured at 7.4 × 5.6 × 3.8 cm over the period of 36 years. The unique presentation of multiple large nodules on the head and extending to other areas of the body may be secondary to socioeconomic challenges. Being in a rural area of the United States, the patient elected to delay healthcare evaluation. The concern with regards to this delay in evaluation could have allowed for lesions to grow further and possible malignant transformation, adding to the uniqueness of this case.

## Data Availability

No data were used to support this study.
